# Exercise Degrades Bone in Caloric Restriction, Despite Suppression of Marrow Adipose Tissue (MAT)

**DOI:** 10.1002/jbmr.3872

**Published:** 2019-10-25

**Authors:** Cody McGrath, Jeyantt S Sankaran, Negin Misaghian‐Xanthos, Buer Sen, Zhihui Xie, Martin A Styner, Xiaopeng Zong, Janet Rubin, Maya Styner

**Affiliations:** ^1^ Department of Medicine, Division of Endocrinology University of North Carolina Chapel Hill NC USA; ^2^ Department of Computer Science University of North Carolina Chapel Hill NC USA; ^3^ Department of Psychiatry University of North Carolina Chapel Hill NC USA; ^4^ Biomedical Research Imaging Center University of North Carolina Chapel Hill NC USA

**Keywords:** BONE‐FAT INTERACTIONS, EXERCISE, MARROW ADIPOSE TISSUE (MAT)

## Abstract

Marrow adipose tissue (MAT) and its relevance to skeletal health during caloric restriction (CR) is unknown: It remains unclear whether exercise, which is anabolic to bone in a calorie‐replete state, alters bone or MAT in CR. We hypothesized that response of bone and MAT to exercise in CR differs from the calorie‐replete state. Ten‐week‐old female B6 mice fed a regular diet (RD) or 30% CR diet were allocated to sedentary (RD, CR, *n* = 10/group) or running exercise (RD‐E, CR‐E, *n* = 7/group). After 6 weeks, CR mice weighed 20% less than RD, *p* < 0.001; exercise did not affect weight. Femoral bone volume (BV) via 3D MRI was 20% lower in CR versus RD (*p* < 0.0001). CR was associated with decreased bone by μCT: Tb.Th was 16% less in CR versus RD, *p* < 0.003, Ct.Th was 5% less, *p* < 0.07. In CR‐E, Tb.Th was 40% less than RD‐E, *p* < 0.0001. Exercise increased Tb.Th in RD (+23% RD‐E versus RD, *p* < 0.003) but failed to do so in CR. Cortical porosity increased after exercise in CR (+28%, *p* = 0.04), suggesting exercise during CR is deleterious to bone. In terms of bone fat, metaphyseal MAT/ BV rose 159% in CR versus RD, *p* = 0.003 via 3D MRI. Exercise decreased MAT/BV by 52% in RD, *p* < 0.05, and also suppressed MAT in CR (−121%, *p* = 0.047). Histomorphometric analysis of adipocyte area correlated with MAT by MRI (R^2^ = 0.6233, *p* < 0.0001). With respect to bone, *TRAP* and *Sost* mRNA were reduced in CR. Intriguingly, the repressed *Sost* in CR rose with exercise and may underlie the failure of CR‐bone quantity to increase in response to exercise. Notably, *CD36*, a marker of fatty acid uptake, rose 4088% in CR (*p* < 0.01 versus RD), suggesting that basal increases in MAT during calorie restriction serve to supply local energy needs and are depleted during exercise with a negative impact on bone. © 2019 The Authors. *Journal of Bone and Mineral Research* published by American Society for Bone and Mineral Research.

## Introduction

Marrow adipose tissue (MAT) accumulation was initially detected in the 1970s by Meunier and colleagues in orthopedic surgical specimens of osteoporotic patients as well as in the setting of normal aging.^1^ These first findings impelled research into the significance of bone marrow adipocytes for skeletal health. MAT, derived from the differentiation of mesenchymal stem cells (MSC) into adipocytes, increases in bone‐fragility states; however, its potential role in promoting bone formation and/or resorption has not been elucidated, despite active investigation.^2^, ^3^, ^4^, ^5^, ^6^, ^7^, ^8^, ^9^, ^10^, ^11^ Understanding of MAT has improved with quantification methods that permit exact investigations, including magnetic resonance spectroscopy (MRS) in humans^12^, ^13^, ^14^, ^15^ as well as osmium‐μCT and MRI with advanced image processing in rodents.^16^, ^17^, ^18^ Recent work established MAT to be suppressed by exercise, in rodents^16^, ^18^ and humans,^19^ suggesting that MAT may function similarly to white adipose tissue in a calorie‐replete state as an energy depot. Moreover, fatty acid β‐oxidation markers rise in bone in the setting of exercise, concomitant with increased bone quantity; this along with research^9^, ^20^ demonstrating the reliance of the osteoblast on β‐oxidation support MAT's role as an energy depot.

In addition to aging‐associated osteoporosis, MAT appears to increase in the fragile bone states of anorexia in humans and caloric restriction in mice. As nutrient stores in caloric restriction wane, gluconeogenesis provides energy^21^, ^22^, ^23^, ^24^, ^25^ and fat stores are mobilized as an alternate fuel source.^26^, ^27^ In late caloric restriction, white fat stores are depleted, highlighting the conundrum of persistent marrow adipocytes.^28^, ^29^ Although marrow fat accumulation in the energy‐depleted state has been shown in humans by marrow aspirate^30^ or MRS^31^ and rodents via histology and MRS,^32^ studies lacked 3‐dimentional valuation of MAT. Prospective caloric restriction studies are unlikely to receive institutional review board approval and thus, in humans, we largely rely on retrospective or cohort studies, limiting data quality. Imaging and bone biopsies in human studies are difficult to obtain, further dictating a need for animal studies. Thus, rigorous measures of MAT response to caloric restriction are needed.

We have shown that MAT increases with overfeeding and decreases during exercise.^17^, ^18^ This supports that MAT functions as an accessible energy depot. In addition to the paucity of data for MAT quantity and localization in states of caloric restriction, its physiology in this setting is poorly understood. The pathologic bone loss due to anorexia/caloric restriction shows a minimal anabolic‐bone response to exercise and maintains a significant fracture risk for years after successful weight gain.^33^, ^34^, ^35^ This stands in contrast to the exercise effect to increase bone formation while decreasing resorption in the calorie‐replete state.^36^, ^37^, ^38^, ^39^, ^40^, ^41^, ^42^, ^43^, ^44^, ^45^ We thus hypothesized that in caloric restriction, MAT's physiologic role differs from the calorie‐replete state. The reports of increased MAT in calorie restriction,^28^, ^29^ combined with increased fracture risk, suggest that the MAT energy depot may be subverted in the energy‐depleted state. Indeed, a high level of physical activity—in combination with caloric restriction—likely results in a decline in overall health,^46^ based on Potzner's constrained energy expenditure model.^47^ Accordingly, we asked if exercise might be harmful to skeletal health in the setting of caloric restriction, simultaneous with exact quantification and characterization of the physiologic response of MAT and bone.

Our findings confirmed that exercise in the setting of a calorie deficit is harmful to bone health. We observed a degradation of bone in exercised, calorically restricted mice. Interestingly, MAT decreased in CR‐exercisers compared with CR, and this was significant, suggesting an alternative purpose for the marrow fat depot in the setting of caloric restriction. Further, reduced sclerostin (*Sost*) and *TRAP* during caloric restriction reflected a low bone turnover state. Both rose during exercise in calorie restriction. Lastly, we noted that *CD36*, responsible for fatty acid uptake, was significantly upregulated in caloric restriction, supplying a prospective mechanism by which MAT expands in this state.

## Materials and Methods

### Animals, diet, and exercise intervention

Procedures were approved by the University of North Carolina Institutional Animal Care and Use Committee. Eleven‐week‐old C57BL/6 (B6) female mice (Jackson Laboratory, Bar Harbor, ME, USA) were housed in controlled light and temperature conditions. Individually housed mice were randomly allocated to an *ad libitum* regular diet (RD) group or a 30% caloric restriction (CR) group for 6 weeks (#D12450J, Research Diets, New Brunswick, NJ, USA, containing 10% of the calories from fat and the corresponding, nutrient‐enriched CR diet, #D15032801). Both RD and CR diets contain 10% of the calories from fat. The CR diet is based on RD but modified as a daily allotment to provide 70% of the caloric intake as well as100% of vitamins and minerals.^48^ Concomitant with dietary intervention, mice were further allocated to voluntary running wheel exercise (E) for 6 weeks as previously described.^16^, ^17^, ^18^ Both RD‐E and CR‐E mice ran during the 6 weeks of wheel access. We did not exclude mice as all runners took part in voluntary running daily. Cyclometers record the daily distance as well as average velocity as in Styner and colleagues.^17^

### Volumetric quantification and imaging of MAT by MRI

Imaging using a 9.4 T horizontal small‐bore MRI scanner was applied to quantify MAT volumetrically.^17^ Briefly, femoral water and fat maps were obtained with a 2‐dimensional RARE imaging sequence with the following parameters: RARE factor = 4, TE = 28 ms, TR = 4000 ms, number of averages = 4, number of slices = 24, slice thickness = 0.5 mm, in‐plane resolution = 100 × 100 μm^2^, matrix size = 130 × 130. As fat and water protons have an NMR frequency separation of 3.5 ppm, a Gaussian‐shaped 90° saturation pulse with a width of 2 ms was applied preceding the RARE sequence to suppress fat or water signal while leaving the other signal unaffected. Fat and water images were acquired by setting the saturation pulse frequency the same as the water and fat frequencies, respectively.

In our processing workflow, we manually subdivided full images containing samples into individual images for each bone. Then, we employed water images to manually contour femoral bone masks using Insight SNAP.^49^ Using these masks, interior bone regions were masked from other image parts in both water and fat maps. Next, a common, study‐specific reference space was established by computing an unbiased average image^50^ from the masked water maps using the ANTS registration software.^51^ Individual water and fat maps were propagated into the common space, where voxel‐wise correspondence allows direct comparison of intensities. Average fat maps for each group were computed in the common space and superimposed on the common, average water image for visualization of group fat maps. Fat map intensities were represented with a colored heat map in 3D Slicer^49^ for visualization (as in Fig. 3 *A*, *B*). For MAT quantification (as in Fig. 3 *C*), a regional label map of the femur was created, excluding cortical bone regions, for the epiphysis, metaphysis, and diaphysis. The femoral head was excluded from the final analysis due to variability in the bone shape/volume between specimens. Intensity‐weighted volume of MAT was quantified via regional fat histograms as in Styner and colleagues.^17^

### Histomorphometry

Fixed and decalcified femurs were imbedded in paraffin, sectioned at 5 μm, and stained with hematoxylin as previously described.^17^, ^52^ Imaging was performed via the Olympus X81 at 4× and 40× magnifications. The 40× magnification images were obtained at the distal femoral growth plate, where lipid content is maximal. ImageJ was used to isolate adipocytes and quantify adipocyte size and number.^17^, ^53^ This process was applied to 3 animals/group and 3 sections/animal, with a minimum of 300 cells analyzed per experimental group. For osteoclast quantification, sections were stained for TRAP with a Fast Green (Sigma, St. Louis, MO, USA, F7252‐5G) background stain. Analysis for osteoclast number was performed using the open source applications Image J and TrapHisto.^54^

### Bone microarchitecture

Bone microarchitecture parameters of the proximal tibial metaphysis and mid‐diaphysis were quantified ex vivo as previously described (resolution = 12 μm, E = 55 kVa, I = 145 μA).^17^, ^55^, ^56^, ^57^

### Real‐time PCR

Quantitative PCR was performed as previously described.^16^, ^18^, ^58^, ^59^, ^60^ Briefly, 1 μg of mRNA from whole tibia was reverse‐transcribed. Ten microliters of cDNA from each experimental condition were pooled and diluted 1:10 to 1:10,000 to generate a 5‐point standard curve. A non‐template control was added to each PCR reaction. Standards and samples were run in duplicate. PCR products were normalized to GAPDH.

### Statistical analysis

Statistical significance was assessed by two‐way ANOVA with correction for multiple comparisons via a Tukey post hoc test (GraphPad Prism 7.0, GraphPad, La Jolla, CA, USA), applying exercise and dietary intervention as analysis variables. Our data sets passed the Shapiro–Wilk normality test. The *p* value cut‐off for significance is defined at less than or equal to 0.05.

## Results

### Caloric restriction attenuates white adipose tissue and body weight

**Figure 1:**
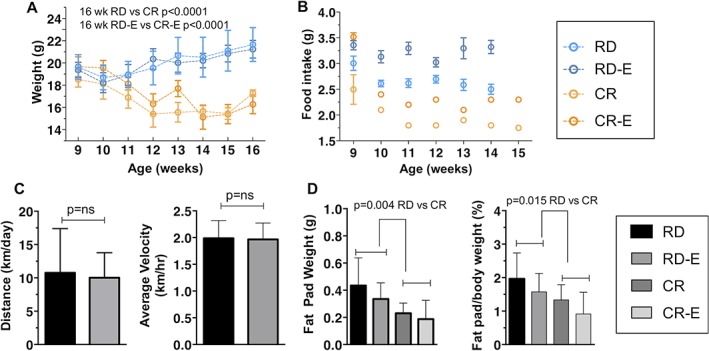
Caloric restriction reduces body and perigonadal fat pad weight. B6 mice after 6 weeks of 30% caloric restriction (CR) versus regular diet (RD) +/− running exercise (E) (4 groups, *n* = 7–10/group). (*A*) Body weight. (*B*) Food intake (g/d). (*C*) Running distance (km/d) and average running velocity (km/hr). (*D*) Fat pad weight. Mean ± SD. Significance by 2‐way ANOVA or *t* test.

To investigate marrow adiposity in caloric restriction and its relevance to skeletal health, B6 mice assigned to a 30% caloric restriction (CR) versus regular diet (RD) were further allocated to voluntary exercise (E) versus sedentary control group. Running distance was similar between the groups (Fig. 1 *C*, RD‐E 10.8 ± 6.6, CR‐E 10.03 ± 3.8, *p* = ns), with individual variability noted, consistent with other rodent studies applying a voluntary running exercise intervention.^61^, ^62^ Running speed was also likewise similar between groups (Fig. 1 *C*).

After 6 weeks, calorically restricted mice weighed 20% less than RD, *p* < 0.0001; exercise did not significantly affect weight (Fig. 1 *A*). The gonadal fat pad weight % was 31% lower in CR compared with RD (Fig. 1 *D*). The fat pad weight was significantly reduced applying diet as the main effect by 2‐way ANOVA (*p* = 0.004 for fat pad weight, *p* = 0.015 for fat pad weight %). Thus, CR mice had demonstrably lower fat pad weights in this analysis. Exercise, when applied as a main effect, failed to significantly affect fat pad weight or fat pad weight %.

### Cortical and trabecular bone is degraded by exercise in caloric restriction

**Figure 2:**
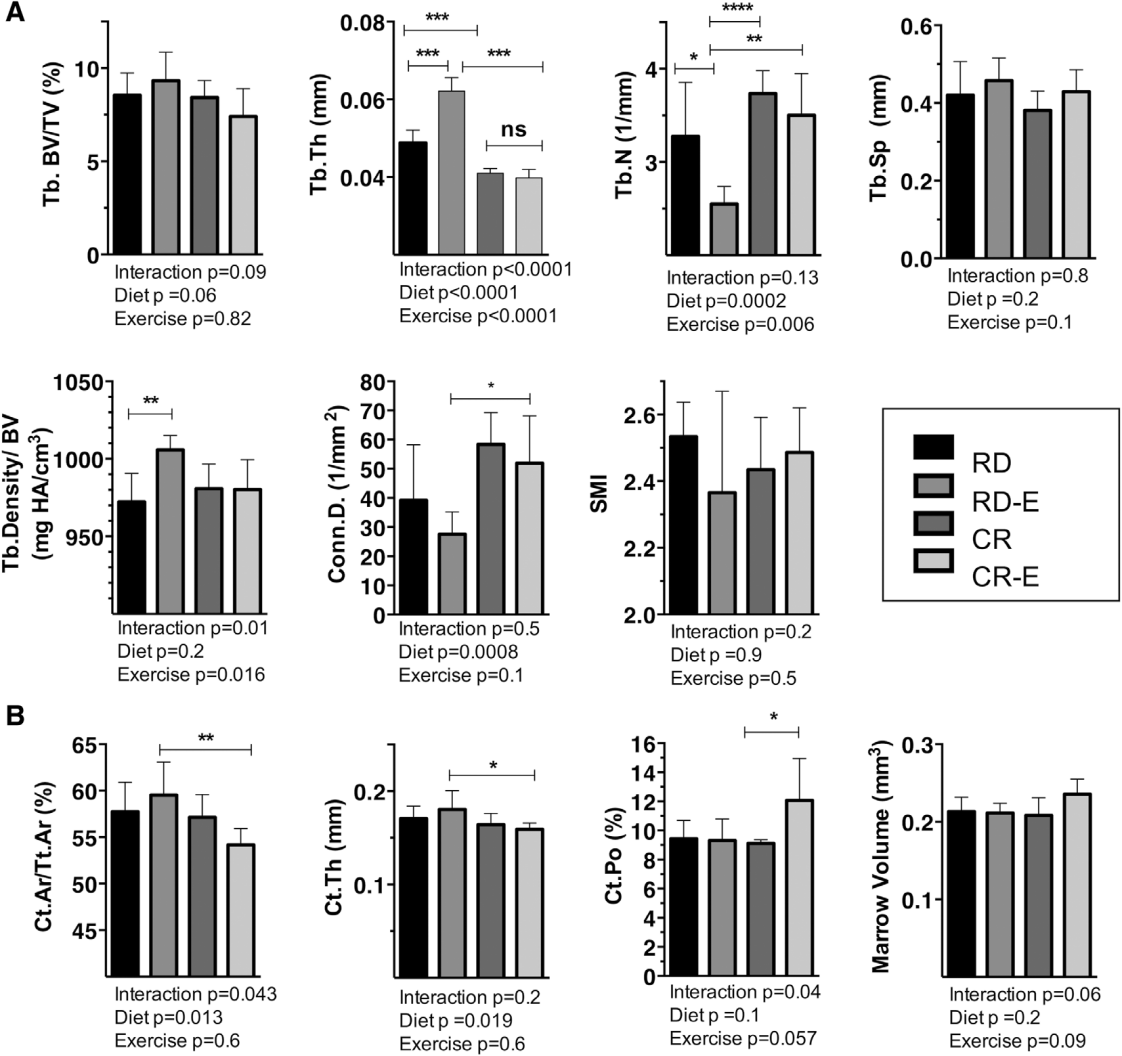
Bone quantity was degraded by exercise in caloric restriction. Tibial bone microarchitecture via μCT in B6 mice after 6 weeks of 30% caloric restriction (CR) or regular diet (RD) +/−running exercise (*E*) (*n* = 7/group). (*A*) Trabecular parameters. (*B*) Cortical parameters. Plots represent means ± SD. Significance by 2‐way ANOVA. For multiple comparisons, *p ≤ 0.05, **p ≤ 0.01, ***p ≤ 0.001****p ≤ 0.0001.

Consistent with preclinical and clinical studies, trabecular microarchitecture measured in the tibia via μCT demonstrated reduced bone quantity in caloric restriction (Tb.Th −16% in CR versus RD, *p* < .001; Tb.Th −45% in CR‐E versus RD‐E, *p* < 0.0001, Fig. 2 *A*). Cortical parameters such as cortical thickness and cortical area fraction were similarly decreased in CR. An anabolic response to exercise in the calorie‐replete RD mice was found, consistent with prior work.^16^, ^17^, ^18^ Specifically, RD mice showed a 27% increase in Tb.Th in response to exercise (*p* < 0.0001, Fig. 2 *A*). In contrast, the calorie‐restricted group demonstrated degraded bone parameters with exercise: reduced trabecular number (−45% CR‐E versus RD‐E, *p* = 0.002) as well as increased cortical porosity (+26% CR‐E versus RD‐E, *p* = .05; +28%, CR‐E versus CR *p* = 0.04, Fig. 2 *B*). Thus, the response of CR bone to exercise veers sharply from the positive anabolic response of RD runners. This suggests that a calorie‐replete state is required for exercise‐induced skeletal anabolism. Moreover, exercise‐induced suppression of MAT may mobilize bone lipid for lipolysis; however, it is unlikely to serve as an energy store for bone formation in CR.

### Exercise reduced marrow adipose tissue volume in the setting of caloric restriction

**Figure 3:**
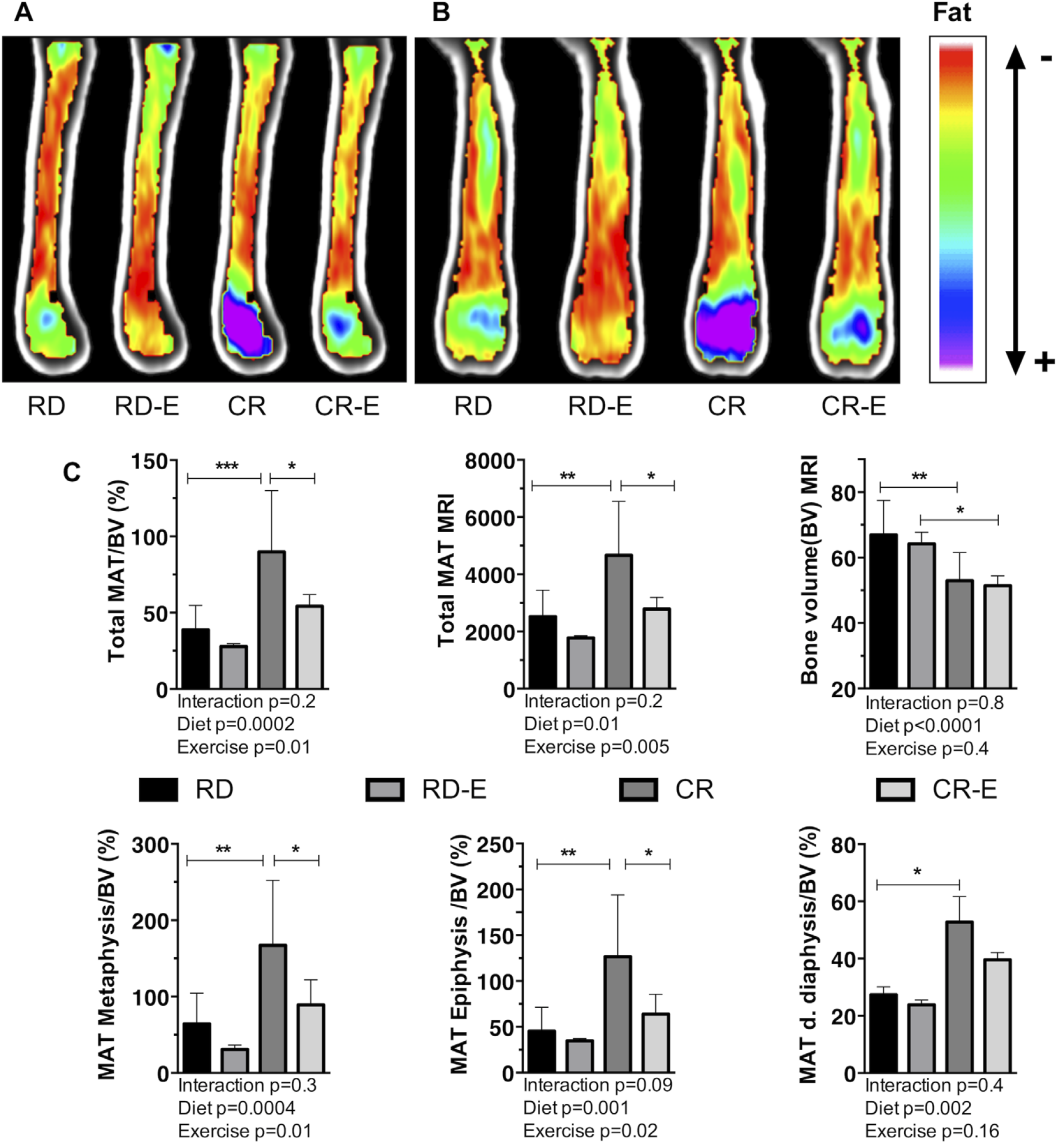
Exercise associated with reduced marrow adipose tissue (MAT), even in the setting of caloric restriction. Average MRI group images (*n* = 6–9/experimental group) in sagittal (*A*), coronal (*B*) planes color labeled for quantity of lipid. B6 mouse femurs analyzed 6 weeks after 30% caloric restriction (CR) or regular diet (RD) +/− running exercise (E) via MRI with advanced image processing. (*C*) Bone volume (BV) and marrow adipose tissue (MAT) quantification via MRI. Mean ± SD. Significance by 2‐way ANOVA. For multiple comparisons, *p ≤ 0.05, **p ≤ 0.01, ***p ≤ 0.001.

We next turned to the question of whether MAT is quantifiably increased in mice in the setting of caloric restriction. We also sought to find if exercise might attenuate MAT in CR as previously shown in a calorie‐replete state.^16^, ^18^ MAT was quantified by means of volumetric magnetic resonance imaging (MRI), which allows separation of the nuclear magnetic resonance signals from water and fat, along with bone masking, allowing a precise quantification of MAT relative to bone volume in murine femurs.^17^ Average group femur MR images (*n* = 6–9/group, Fig. 3 *A*, *B*) display the distribution of MAT in the femur with a higher MAT signal in the metaphysis/epiphysis in the calorically restricted group. As expected, and corresponding to body weight measurements, bone volume (BV) measured 21% lower in CR versus RD (*p* = 0.04) and 19% lower in CR‐E versus RD‐E (*p* = 0.03, Fig. 3 *C*). Total femoral MAT/BV in CR, in contrast to white adipose tissue, increases (+132%, *p* = 0.0009), with individual regions such as the distal epiphysis, metaphysis, and diaphysis demonstrating a significant increase as well (Fig. 3 *C*). Notably, while the MAT content increased with CR in multiple regions, it was most evident in the metaphysis (+159%, *p* = 0.003, Fig. 3 *C*). In response to exercise, whole bone MAT/BV diminished significantly in both experimental groups (RD‐E versus RD: –28%, CR‐E versus CR: −92%, *p* = 0.01 for an exercise effect, Fig. 3 *C*), akin to prior findings demonstrating exercise‐induced diminution of MAT in non‐calorically restricted states.^16^, ^18^ In terms of regional analysis, metaphyseal MAT/BV was particularly responsive to exercise (− 52% in RD‐E versus RD and −121% in CR‐E versus CR, *p* = 0.01 for an exercise effect) (Fig. 3 *C*). Adipocyte size vis histology correlated with the MRI data: −48% in RD‐E versus RD and −20% in CR‐E versus CR, *p* = 0.006 for an exercise effect (for correlation R^2^ = 0.6233, *p* < 0.0001) (Fig. 5 *A–C*).

### Exercise increases markers of resorption in caloric restriction

**Figure 4:**
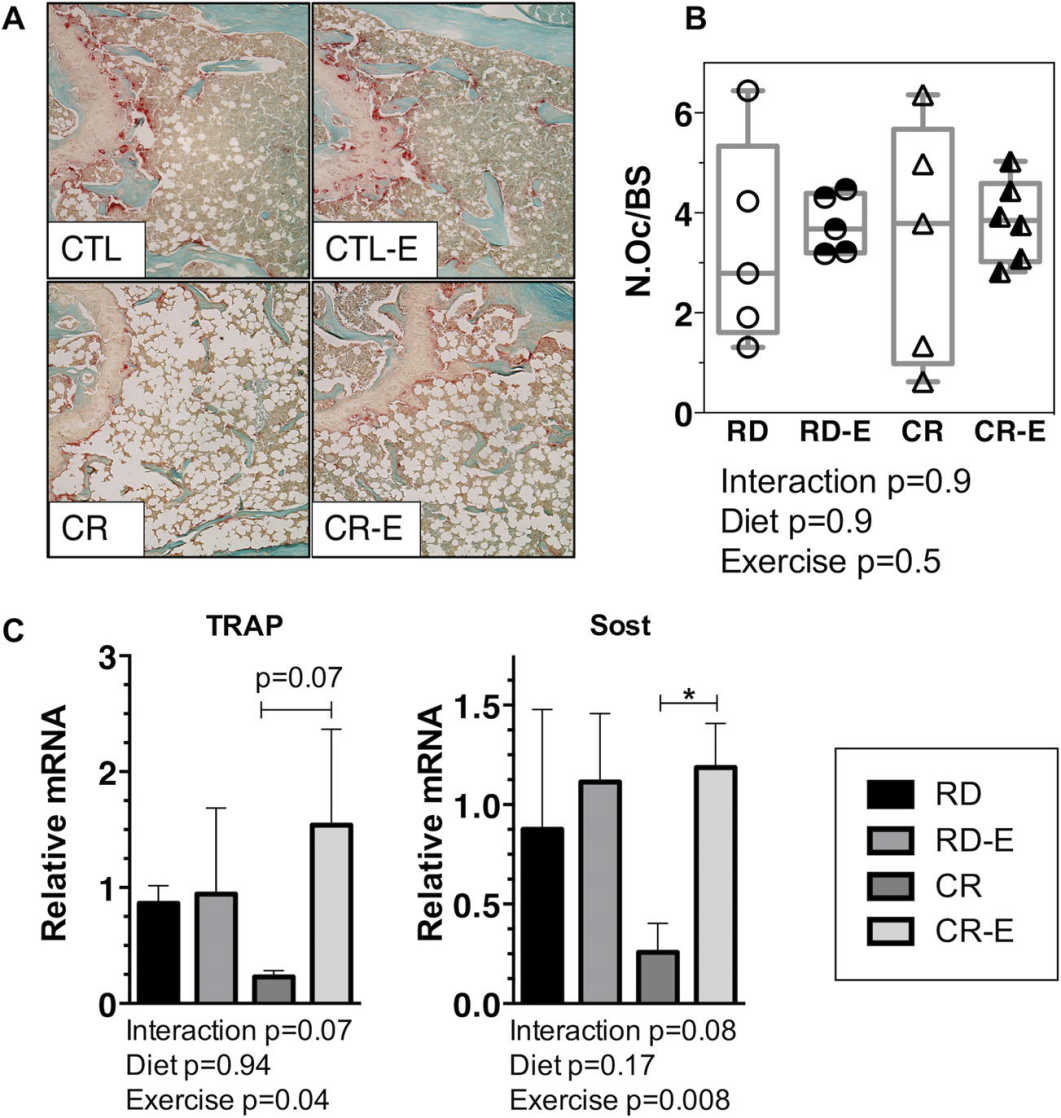
Exercise, in the setting of caloric restriction, attenuates markers of bone resorption. (*A*) TRAP (red) stain of osteoclasts in representative histologic sections of tibias after 6 weeks of 30% CR +/− exercise. (*B*) Osteoclasts quantified via semi‐automated histomorphometry in mouse femurs (*n* = 4–6/ group) with individual mice plotted. (*C*) Tibial mRNA via qPCR (*n* = 4/group). Means ± SD. Significance by 2‐way ANOVA between experimental groups. For multiple comparisons, *p ≤ 0.05.

Next, we queried whether bone resorption might be involved in the increased bone degradation noted in CR exercisers. Osteoclast number was quantified via static histomorphometry (*n* = 4–6 mice /group) normalized to the bone surface (N.Oc/BS). The N.Oc/BS analysis shows no statistically significant difference between the groups (Fig. 4 *B*). The analysis of the variance does show that more of the variance is accounted for by exercise status than by diet. It is possible that the high variability of N.Oc/BS in sedentary groups did not permit statistically significant differences to emerge in this analysis and that a larger number of animals would be required to definitively quantify a difference in N.Oc/BS.

In our qPCR analysis, *TRAP* mRNA, which was down 73% in CR versus RD, rose 150% in CR‐E versus CR (*TRAP* mRNA, *p* = 0.04 for exercise effect). Sclerostin or *Sost* mRNA similarly was reduced 79% in CR versus RD and rose 115% in CR‐E versus CR (*p* = 0.009 for exercise as main effect, Fig. 4 *C*). Thus, mRNA data demonstrates an increase in markers of bone resorption in the exercise groups, although staining for osteoclasts did not reach significance. Because tibias were used for PCR and microarchitecture and femorae for MAT by MRI and histomorphometry, results might not be generalizable to other long bones, and correlation of outcomes between disparate bones requires care.

### Bone fatty acid uptake underlies MAT expansion in caloric restriction

**Figure 5:**
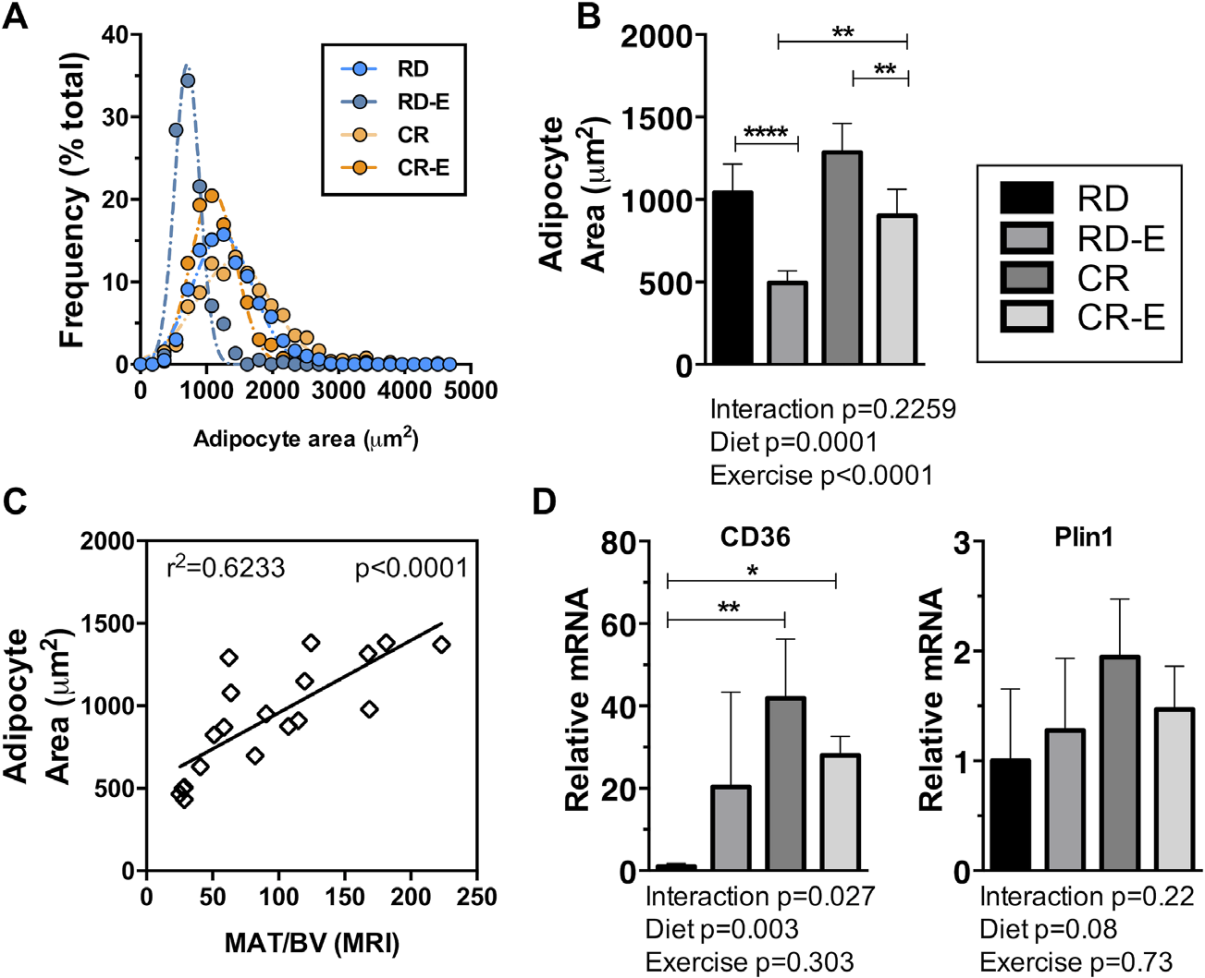
Caloric restriction, exercise attenuation of marrow adipocyte area, marker of fatty acid metabolism. Histomorphometric analysis performed on 3 sections per mouse (*n* = 5–6) with a minimum of 300 cells analyzed for each experimental group. (*A*) Marrow adipocyte area histogram. (*B*) Marrow adipocyte area. (*C*) qPCR on mRNA from tibias (*n* = 4). (*D*) Linear correlation plot of adipocyte area versus MAT/BV via MRI analysis; both performed in distal femoral metaphysis. Plots demonstrate means ± SD. Significance by 2‐way ANOVA between experimental groups. For multiple comparisons, *p ≤ 0.05, **p ≤ 0.01, ***p ≤ 0.001, ****p ≤ 0.0001.

Adipocyte area increased in CR compared with RD (Fig. 5 *B*) and correlated with MAT by MRI (correlation R^2^ = 0.6233, *p* < 0.0001), demonstrating significant fat accumulation in CR‐bone. We sought to investigate potential pathways by which marrow adipocyte accumulation occurs in this setting. Lipid droplet markers such as *perilipin 1* or *Plin1* was highest in the CR group (Fig. 5 *C*). Interestingly, caloric restriction significantly increased *CD36*, a marker of fatty acid uptake (+4088%, *p* < 0.01 CR versus RD), suggesting a mechanism for lipid accumulation in the bone in CR. Exercise attenuated *CD36* in CR consistent with exercise induced suppression of MAT in this setting by MRI and histology (Fig. 5 *C*, −1394% CR‐E versus CR). Additionally, in RD‐exercisers, fatty acid uptake marker *CD36* rose while in CR‐exercisers it declined, suggesting a divergent bone metabolic profile in these states that may dictate lipid uptake and utilization.

## Discussion

Adipocytes in the bone marrow serve critical roles, regulating homeostasis in hematopoietic niches^63^, ^64^ and storing energy for use during exercise.^17^ An increase in marrow fat is found during conditions associated with osteoporosis—aging and estrogen deficiency^12^—and the same has been suggested in the unique bone fragility that accompanies caloric restriction.^31^, ^32^, ^65^ Here we quantified marrow adiposity with advanced image analysis during caloric restriction with an added stressor of exercise. By means of 9.4 T MRI 3D images, we demonstrated that marrow fat relative to bone volume increased during caloric restriction, most notably in the femoral metaphysis when compared with other regions of the femur. Although peripheral adipose depot size decreased due to energy utilization during caloric restriction, the size of marrow adipocytes increased. As such, adipocyte hypertrophy and significant lipid accumulation occurred in bone in the presence of an energy‐depleted state. Along with the increase in bone marrow adiposity, we demonstrated suppressed bone turnover markers in sedentary calorie‐restricted mice. Importantly, when CR mice exercised, marrow fat declined, and bone turnover markers increased. In sum, exercise requires energy for anabolism, and exercise in the absence of stored fat might result in sacrificing tissue to supply needed calories.

Our finding of increased bone turnover markers in exercised, calorically restricted mice is clinically important, as exercise is frequently proposed as a form of therapy for patients with bone fragility. Bone resorption in the calorically restricted state likely depends on several factors such as sex, severity of CR, duration of CR, and physical activity intensity. A human study of anorexic girls revealed reduced urinary N‐terminal telopeptide (NTX), a bone resorption marker,^66^ suggesting decreased bone turnover consistent with findings in our CR mice. Hypoestrogenism is part of anorexia in women^65^ and caloric restriction in female mice^67^ though, estrogen therapy has displayed variable efficacy in anorexia for bone density endpoints.^68^, ^69^, ^70^ A limitation of our study is the lack of estradiol measurements. Since exercise exacerbates hypoestrogenism,^71^ hormonal status might contribute to the deterioration of bone as well as to likely increased resorption in CR‐exercisers.

The mechanisms by which MAT accrues in the calorie‐restricted state continue to be an area of active investigation.^3^, ^72^ Increased glucocorticoids, Pref‐1, and MAT‐derived adiponectin^28^, ^73^, ^74^ as well as low IGF‐1 and leptin^28^, ^32^, ^75^, ^76^, ^77^ have been associated with MAT in CR; however, causality has not been established for these factors in driving MAT nor in the skeletal deterioration of CR. Systemic sclerostin inhibition was shown to reduce marrow adipocytes.^78^ Our data show bone *Sost* expression decreased during calorie restriction, potentially part of the mechanism underlying decreased bone turnover. Interestingly, *Sost* increased with exercise, whereas MAT decreased. Future investigation is required to determine the role of *Sost* in MAT accumulation.

We found a significant increase in fatty acid translocate/CD36 in bones of calorically restricted animals, suggesting an augmented cellular uptake of fatty acids.^79^ A study of CD36 knockout mice revealed reduced bone quantity, suggesting that CD36 is important for skeletal health.^80^ CD36 possesses cellular functions related to use of fat calories including functioning as a receptor for oxidized low‐density lipoprotein (LDL)^81^ as well as regulating fatty acid uptake in skeletal muscle and cardiomyocytes. CD36 upregulation in muscle can occur in the setting of increased dietary fat availability, driven by AMP‐activated protein kinase (AMPK) activation.^82^ During starvation, CD36 is upregulated in muscle and understood to be a fundamental regulator of muscle's *metabolic flexibility*, reducing the tissue's reliance on glucose and increasing the utilization of fatty acids for energy.^83^ in vitro experiments exhibited a preference for glucose as an energy source in cultured osteoblasts.^84^ It is unclear whether osteocytes, marrow adipocytes, and their progenitors rely on fatty acids or glucose in the calorie‐restricted state and exercised states. Recent human metabolomic data obtained after 10 days of starvation points to a shift from carbohydrate to fatty acid metabolism.^27^ Here the increase in *CD36* in the bone of calorie‐restricted mice associates with the rise in MAT and may provide a metabolic mechanism for MAT accumulation despite the energy‐deficient state.

Exercise consumes calories from several substrates, including carbohydrates and fat, to supply energy for muscle and skeletal anabolism.^17^, ^85^ The MAT present in the CR state, and its apparent utilization during exercise, however, did not support bone formation. In fact, calorie‐restricted exercisers began with low‐turnover markers compared with calorie‐replete animals and responded to exercise with rises in resorption markers *Sost* and *Trap* in CR bone. As such, exercise‐induced cortical porosity and marrow area increases in CR‐E, along with diminished cortical thickness and cortical bone fraction, indicate bone was quantitatively reduced. Although cortical porosity is not a direct measure of strength, it is distinctly associated with reduced strength^86^, ^87^, ^88^ and thus reflects probable diminution of bone quality as well. Notably, low‐magnitude mechanical stimulation (LMMS), an exercise mimic, was similarly found to increase resorption in human anorexia, along with reduced markers of bone formation.^89^ In accordance with our findings in mice, Swift and colleagues demonstrated increased bone resorption in food‐restricted, exercised rats.^67^ Although studies have shown that bone loss due to anorexia in humans^33^ and caloric restriction in mice^34^, ^67^ is not ameliorated by exercise, ours is the first to show further degradation of bone quantity with voluntary exercise. The voluntary exercise intervention applied is distinct from prior studies, which applied forced running; indeed, mice ran despite being calorie restricted and in spite of increased energy needs. Southmayd and colleagues showed that for exercising humans, bone loss and resorption were higher in the energy‐deficient state.^90^ Our outcome that exercise can be harmful to the skeleton during calorie restriction is additionally in line with Pontzer's constrained energy expenditure model that suggests increasing quantities of physical activity are not necessarily additive with regard to improved health and may be constrained based on nutrient availability.^46^, ^47^

In conclusion, during the calorie‐replete state, exercise induces skeletal anabolism and alters skeletal architecture through effects on a multiplicity of cells.^91^ In the calorie‐replete state, data support that energy stored in marrow adipocytes are utilized for energy during exercise.^2^ In striking contrast, we demonstrate here that exercise appears to be harmful to bone during calorie restriction, in congruence with clinical data.^90^ Thus, despite MAT expansion in caloric restriction, this fat depot might not be harnessed to support energy needed to sustain bone anabolism as well as prevent bone resorption in the energy‐restricted sedentary and exercised states.

## Disclosures

All authors state that they have no conflicts of interest.
